# Deep learning-driven hybrid model for short-term load forecasting and smart grid information management

**DOI:** 10.1038/s41598-024-63262-x

**Published:** 2024-06-14

**Authors:** Xinyu Wen, Jiacheng Liao, Qingyi Niu, Nachuan Shen, Yingxu Bao

**Affiliations:** 1 School of Management Science and Engineering, Shandong University of Finance and Economics, Jinan, 250014 China; 2 School of Economics and Management, Hubei Institute of Automobile Technology, Shiyan, 442002 China; 3 College of International Education, Henan Normal University, Xinxiang, 453007 China; 4 Chinese Academy of Fiscal Science, Beijing, 100142 China; 5 School of Politics and Public Administration, Zhengzhou University, Zhengzhou, 450001 China

**Keywords:** Smart grid, Power load forecasting, Energy information management, Deep learning, Temporal convolutional network, Attention mechanism, Energy science and technology, Engineering

## Abstract

Accurate power load forecasting is crucial for the sustainable operation of smart grids. However, the complexity and uncertainty of load, along with the large-scale and high-dimensional energy information, present challenges in handling intricate dynamic features and long-term dependencies. This paper proposes a computational approach to address these challenges in short-term power load forecasting and energy information management, with the goal of accurately predicting future load demand. The study introduces a hybrid method that combines multiple deep learning models, the Gated Recurrent Unit (GRU) is employed to capture long-term dependencies in time series data, while the Temporal Convolutional Network (TCN) efficiently learns patterns and features in load data. Additionally, the attention mechanism is incorporated to automatically focus on the input components most relevant to the load prediction task, further enhancing model performance. According to the experimental evaluation conducted on four public datasets, including GEFCom2014, the proposed algorithm outperforms the baseline models on various metrics such as prediction accuracy, efficiency, and stability. Notably, on the GEFCom2014 dataset, FLOP is reduced by over 48.8%, inference time is shortened by more than 46.7%, and MAPE is improved by 39%. The proposed method significantly enhances the reliability, stability, and cost-effectiveness of smart grids, which facilitates risk assessment optimization and operational planning under the context of information management for smart grid systems.

## Introduction

The smart grid, playing a pivotal role in the development of contemporary power systems, relies on information technology for real-time monitoring, management, and control of the power system. As a critical element of smart grid management, short-term load forecasting is inseparable from the stability, efficiency, and reliable operation of the power system. It guarantees the safe operation and accurate planning of the power system, playing an indispensable role in risk assessment and operational planning for smart grid energy management^1,2^. However, there are various uncertainties affecting electricity load forecasting, such as load fluctuations, weather conditions, and holidays^2^. These challenges are manifested in the co-existence of trends and periodicity, which is compounded by significant stochasticity and volatility. This increases the difficulty in achieving accurate short-term load forecasting^3,4^.

In recent years, there have been some noteworthy breakthroughs made in deep learning technology to address these challenges and enhance the accuracy of load forecasting. Through the construction and training of multi-layer neural networks, deep learning algorithms can extract features from extensive historical load data autonomously, thereby capturing the nonlinear patterns and regularities of load behavior^5,6^. Currently, various traditional deep learning models, have been applied to load forecasting. Some work^4,7–10^ have conducted research on short-term load forecasting models based on algorithms such as Autoencoder, Generative Adversarial Network, Variational Autoencoder, Long Short-Term Memory Attention Network and so on. Below is a brief description of five relatively innovative models:

- Autoencoder: Autoencoder is an unsupervised learning model that learns a compact representation of data through an encoding and decoding process. It can be used for feature extraction and dimensionality reduction. Proposed work could involve integrating it with other models or introducing attention mechanisms to improve load forecasting accuracy^4^.
- Generative Adversarial Networks (GAN): GAN is a generative model that learns the distribution of data and generates realistic synthetic data through adversarial training between a generator and a discriminator. Proposed work could involve utilizing GAN-generated synthetic data to increase the diversity of training samples and improve the generalization capability of load forecasting ^7^.
- Variational Autoencoder (VAE): VAE is a generative model that learns the latent distribution of data and has good sampling capabilities. In load forecasting, VAE can be used to generate additional samples and explore data uncertainty. Proposed work could involve utilizing VAE-generated samples to increase the diversity of training data and considering data uncertainty to enhance the reliability of load forecasting^8^.
- Long Short-Term Memory Attention Network (LSTM-Attention): LSTM-Attention network combines LSTM models with attention mechanisms, capturing long-term dependencies and important features in time series data. Proposed work could involve improving the network structure, optimizing training strategies, and enhancing the focus on key features to improve load forecasting accuracy and stability^10^.
- Transformer Model: The Transformer model introduces self-attention mechanisms, enabling parallel processing of sequence data and exhibiting strong modeling and representation learning capabilities. Proposed work could involve applying the Transformer model to load forecasting, learning the correlations and important features in sequence data to improve prediction accuracy. Additionally, research could focus on optimizing efficiency and scalability of the Transformer model to accommodate large-scale load data processing requirement^11^.

However, existing forecasting methods face various limitations in handling large-scale, high-dimensional energy information and capturing the spatiotemporal characteristics and long-term dependencies of load data. This paper proposes an innovative approach to improve the accuracy and reliability of short-term electricity load forecasting in smart grids, providing valuable insights for the further development of smart grid technologies. It also offers beneficial references for the future development and management of power systems, aiming to pursue a sustainable and efficient energy future. The contribution of this work can be summarized as four points below:

1. Innovative approach: In this study, a predictive model based on a hybrid deep learning approach is introduced, which combines GRU, TCN, and attention mechanism to enhance the accuracy of load forecasting. It is purposed to effectively capture the complex dynamics and long-term dependencies in power data, for more accurate and efficient electricity load forecasting.
2. Training and Evaluation on Multiple Datasets: The model is subject to training and evaluation on four public datasets, namely GEFCom2014, ERCOT Load, AEMO Load, and NYISO. Through the experiment conducted on these datasets, the performance of the algorithm is evaluated, demonstrating its universality and robustness.
3. Applicative results: The experimental results demonstrate the superiority of the proposed method in short-term load forecasting, particularly in capturing spatiotemporal characteristics and accurately predicting future load variations. These findings highlight the significance of the approach in addressing the challenges of load forecasting and advancing the development of intelligent power systems.
4. Long-Term Dependency Modeling: This approach relies on the TCN algorithm to capture the long-term dependence in load sequences. Additionally, the attention mechanism is adopted to weight different segments of the load sequence in real time, highlighting the contribution of essential information. This strategy enhances the performance of the model in capturing the trends and periodic features in load sequences, thus improving predictive accuracy and stability. Through the heightened focus on critical load features, predictive outcomes are further improved.

By accurately predicting electricity demand, the proposed method can facilitate efficient power supply planning, promote the integration of renewable energy sources, and contribute to the overall sustainability and efficiency of the power system.

## Related work

The three most relevant directions include Time Series Analysis and Forecasting, application of Deep Learning in Power Load Forecasting and leveraging Data from Smart Grids and AMI.

1. Time Series Analysis and Forecasting: serves as the foundational and traditional approach for power load forecasting. Given that power load data is intrinsically time-related, time series analysis methods, such as autoregressive models, moving average models, and autoregressive moving average models, become pivotal in this domai^12,13^.
2. Application of Deep Learning in Power Load Forecasting: With the evolution of deep learning technologies, models like LSTM, GRU, and TCN, as previously mentioned, are extensively applied to handle time series data because they can grasp intricate patterns and long-term dependencies within the data^14,15^.
3. Leveraging Data from Smart Grids and AMI: With the advancement of smart grid technologies, there's a proliferation of high-resolution, high-dimensional energy information data. This demands the development of new techniques and algorithms to process such data, enhancing forecasting accuracy and efficiency^16,17^.

### Time series analysis and forecasting

Time Series Analysis refers to the collection of methods used to analyze time-ordered datasets to extract meaningful patterns, correlations, and trends. In the realm of power load forecasting, the goal is often to predict future electricity demand based on past data. Predicting these fluctuations helps utilities better manage resources, reduce costs, and maintain a stable power supply^12,13^. Application in Power Load Forecasting:

- Time series data, like electricity demand, often have underlying structures: trend (long-term movement), seasonality (cyclical fluctuations), and noise (random variations). Decomposition helps isolate these components for a clearer view of underlying patterns.
- Such as seasonal decomposition can highlight increased electricity demand during specific months or times of the day^18^.
- Autoregression (AR) models leverage the relationship between an observation and a number of lagged observations (previous time steps). In AR modeling, today's power load might be predicted based on the load from the past few day^19^.
- Moving Average (MA) models predict the variable of interest using a linear combination of residual errors from previous time points. If there were unexpected spikes in power usage yesterday due to unscheduled industrial activity, MA models can account for this anomaly when forecasting today's load^20^.
- Autoregressive Integrated Moving Average (ARIMA) combines AR and MA methods and can address time series data that is non-stationary (data that has statistical properties changing over time) by differencing. It might be applied to power load data that has both trend and seasonality^21^.
- Exponential Smoothing consider both the trend and seasonality. It provides weight to observations, with more recent observations typically receiving greater weight. If there's a gradual increase in power consumption during winter mornings, exponential smoothing can account for both this increasing trend and the daily morning spike^22^.

Time series models, particularly simpler ones like AR and MA, offer clear insights into the factors influencing forecasts. By combining methods (like ARIMA), time series analysis can handle a wide range of data characteristics. These models are computationally less intensive compared to deep learning models. However, many time series methods require data to be stationary, which might not always be the case for power load data with evolving trends and patterns. Meanwhile, AR-based models are heavily dependent on lagged values, which might not always capture longer-term patterns. While basic models are interpretable, advanced combinations (e.g., SARIMA) can become complex and harder to intuitively grasp.

Time Series Analysis offers a suite of powerful tools for power load forecasting. While they provide a robust starting point and are adept at capturing short-term fluctuations, they might sometimes struggle with long-term trends and non-linear patterns. With the advent of machine learning and deep learning, hybrid approaches are becoming popular, where traditional time series models are combined with advanced algorithms to achieve higher accuracy in predictions.

### Application of deep learning in power load forecasting

Deep learning, a subset of machine learning, has gained significant attention in recent years due to its prowess in handling vast amounts of data and extracting complex patterns. Within the domain of power load forecasting, deep learning techniques offer new avenues to model and predict electricity consumption, addressing many challenges inherent in traditional forecasting methods^14,15^. Application in Power Load Forecasting include:

- Recurrent Neural Networks (RNNs): RNNs are designed to recognize patterns in sequences of data. They maintain a ‘memory’ of past information, which is useful for tasks where temporal dynamics and past information play crucial roles. If a consistent increase in power consumption occurs every weekday at 9 am due to industrial activity, an RNN can detect this pattern and forecast accordingly^23^.
- LSTM and GRU: These are advanced forms of RNNs which can capture long-term dependencies in time series data, something traditional RNNs struggle with due to the vanishing gradient problem. In regions where electricity usage patterns change with seasons, LSTMs can be useful in recognizing patterns that span month^24^.
- Convolutional Neural Networks (CNNs): Originally designed for image processing, CNNs can be used for power load forecasting by treating the time series data as a one-dimensional 'image'. CNNs excel in identifying local and shift-invariant features. It can detect localized patterns such as the consistent evening spike in electricity usage across household^25^.
- Hybrid Models: Combining deep learning models or integrating them with traditional methods can lead to superior forecasting performance. A model might use CNN to extract patterns from power load data and then feed this information into an LSTM to account for longer-term dependencies (v 2022).

Deep learning models, especially LSTMs and GRUs, can capture intricate long-term dependencies and non-linear relationships in the data. It can effectively process vast amounts of data, making them suitable for applications with big data streams. The flexibility of deep learning architectures allows for easy integration and hybridization with other models, improving forecasting performance. However, training deep learning models requires significant computational power and can be time-consuming. Given their complexity, deep learning models can easily overfit to training data, leading to poor generalization to new data unless properly regularized. In comparison to traditional time series models, it can act as “black boxes”, making it challenging to understand and interpret their decision-making processes.

The incorporation of deep learning in power load forecasting has brought about a paradigm shift, allowing for the modeling of more intricate patterns and relationships in electricity consumption data. While they come with their own set of challenges, their potential to improve forecasting accuracy, especially when used judiciously in conjunction with traditional methods, is undeniable. As computational capabilities continue to advance and as algorithms evolve, the role of deep learning in power load forecasting is poised to become even more central.

### Leveraging data from smart grids and Advanced Metering Infrastructure (*AMI*)

With the increasing global demand for renewable energy and efforts towards grid modernization, the importance of smart grid technologies has also grown. Among them, AMI is a crucial component of the smart grid, providing real-time and granular energy usage information to utility companies and consumers^16,17^. The applications of AMI include demand response and load adjustment, where real-time data enables utility companies to better predict electricity demand and supply, dynamically adjust electricity prices, and incentivize users to consume electricity during non-peak hours. AMI allows for quick fault location, speeding up repair times and enabling automatic power redistribution, thus reducing outage durations. AMI provides better access and management of distributed energy sources like solar and wind energy. Users can access detailed data about their own electricity consumption habits and make more energy-efficient choices based on that information^26^.

The advantages of AMI include improved grid efficiency, as real-time data allows for more flexible and efficient resource allocation, reducing energy waste. Additionally, fast fault detection and restoration can minimize outage durations and enhance power reliability. Optimizing energy distribution and encouraging energy conservation can contribute to carbon emission reduction. Furthermore, users can have real-time visibility into their electricity consumption and adjust their usage behaviors accordingly, enhancing the user experience. However, a major disadvantage is the high cost associated with the implementation and maintenance of AMI systems, requiring substantial financial investments. Additionally, the vast amount of personal electricity usage data generated by AMI may raise privacy concerns. Furthermore, any system connected to the internet faces the risk of cyberattacks, which could result in grid failures or data manipulation.

Existing forecasting methods face various limitations in handling large-scale, high-dimensional energy information and capturing the spatiotemporal characteristics and long-term dependencies of load data. Recently, there has been growing attention to research that combines multiple deep learning methods to fully leverage their respective strengths and characteristics, aiming to further improve the accuracy and efficiency of load forecasting^27–29^. For instance, Chen et al.^30^ proposed a short-term load forecasting model based on the CEEMDAN-IGWO-GRU hybrid algorithm. This algorithm combines complete ensemble empirical mode decomposition with adaptive noise and utilizes IGWO and GRU for the decomposition and prediction of load signals, demonstrating great potential in establishing load forecasting models. Tan et al. ^31^ proposes a novel forecast scenario-based robust operation model specifically tailored to the integration of rural energy systems with greenhouses, to address the uncertainties associated with electric load and wind power generation.

## Methodology

### Overview of our network

**Figure 1:**
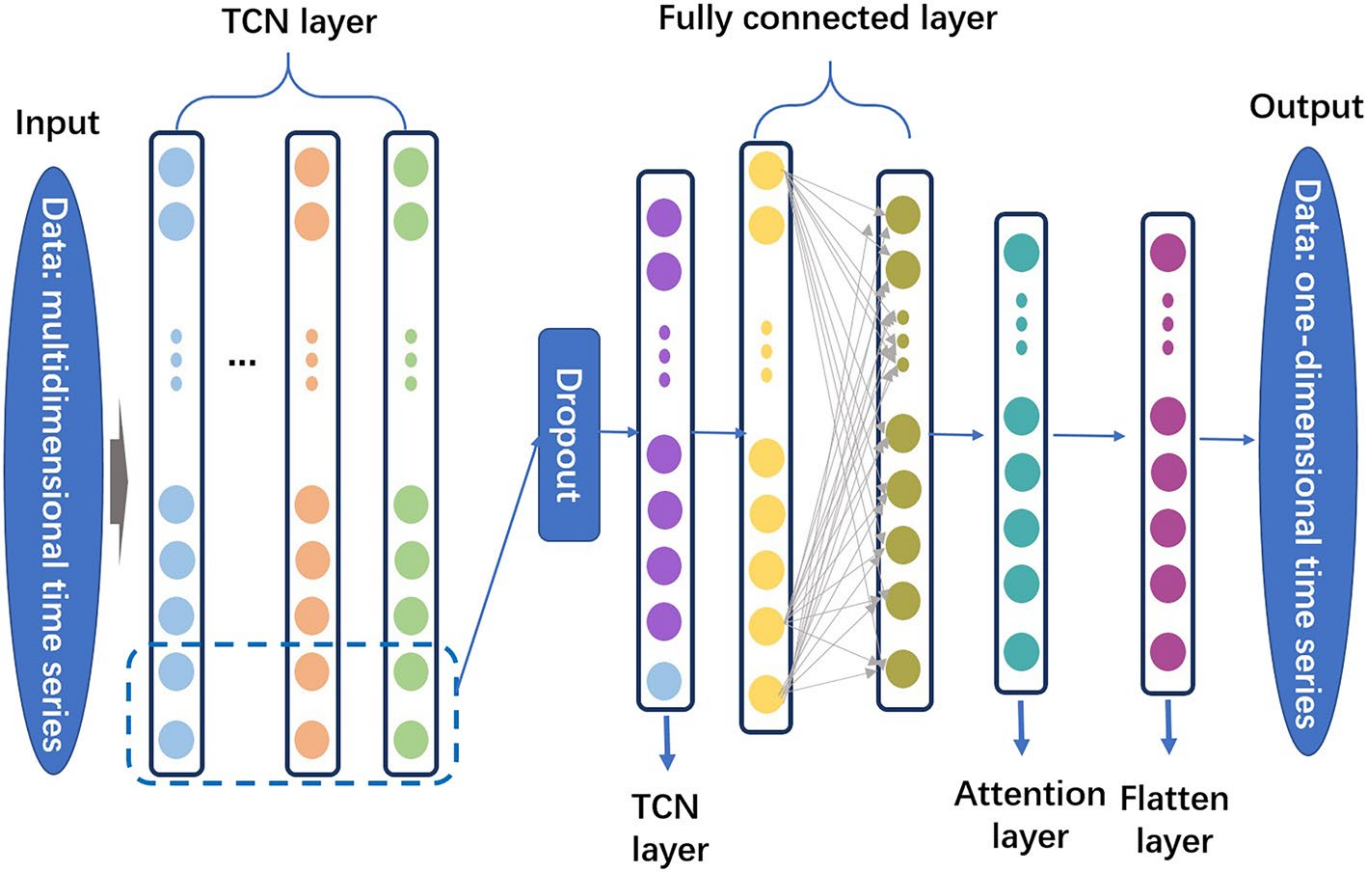
The overall framework diagram of the proposed model.

This paper proposes a hybrid algorithm that combines GRU, TCN and attention mechanism to address the challenges in short-term power load forecasting and energy information management. The algorithm leverages the advantages of GRU in capturing long-term dependencies in time series data, TCN’s ability to extract temporal features, and the attention mechanism's capability to dynamically adjust the importance of different features, thereby improving the accuracy and reliability of predictions. Figure 1 represents the overall framework diagram of the proposed model.

The overall process of the method implementation is as follows:

1. Data Preparation: Collect historical load data and other relevant features, including weather data and holiday information. Preprocess the data by handling missing values and performing normalization.
2. Feature Engineering: In this stage, consider the specific characteristics of the load forecasting task. Extract and select features from the data based on domain knowledge and experience to capture the relevant aspects of load forecasting. Various methods such as statistical features, frequency domain features, or time domain features can be employed. This targeted feature engineering enhances the model's predictive performance.
3. Model Construction and fusion: By combining GRU, TCN and attention mechanisms, the model can capture features and patterns in load data from different perspectives to reduce the impact of uncertainty on prediction results.

Firstly, build a hybrid structure combining GRU and Temporal TCN, leveraging the strengths of both to improve the model’s ability to capture load data. GRU is utilized to capture long-term dependencies in time series data, while TCN focuses on learning local patterns and features in load data.

Secondly, introduce an attention mechanism to prioritize the input components most relevant to the load forecasting task. By learning weight allocations, the attention mechanism enhances the model's focus and predictive accuracy, enabling it to emphasize the features and time periods with the greatest impact on load variations.

Model training

Split the prepared dataset into training, validation, and testing sets.

Train the hybrid model using the training set by minimizing the disparity between the predicted outputs and the actual load demand. Optimization algorithms such as gradient descent can be utilized to update the model parameters.

- Monitor performance metrics on the validation set during the training process to determine the optimal model parameter settings.

5. Uncertainty modeling: During model training and prediction processes, it is important to consider modeling of uncertainty factors. Uncertainty in load data can be modeled by introducing stochasticity or Monte Carlo methods. This obtains a range of possibilities to assess the reliability of the load forecast results and provide a probability distribution or confidence interval.

6. Forecast error analysis and Model Evaluation: By comparing the error between predicted and actual values, the reliability and accuracy of the model can be assessed. At the same time, analysis of errors can identify potential sources of uncertainty and improve model performance.

Evaluate the trained model using the testing set by computing error metrics such as root mean square error (RMSE) and mean absolute error (MAE) between the predicted results and the actual load demand. For load prediction, input the latest data into the trained model to obtain the prediction results.

7. Load forecasting and energy management: Utilize the trained model to predict future load, including short-term and medium-term forecasts. Adjust and optimize energy supply and demand based on the prediction results, such as adjusting power generation plans and managing energy storage.

8. Experimental validation and application: Validate the method using real-world data, assessing its accuracy, reliability, and efficiency. Apply the method to actual energy management systems, enabling practical load forecasting and energy information management.

Through this process, the hybrid algorithm effectively captures the spatial and temporal characteristics of power load data, leading to high-precision short-term load forecasting.

### Gated Recurrent Unit (GRU)

**Figure 2:**
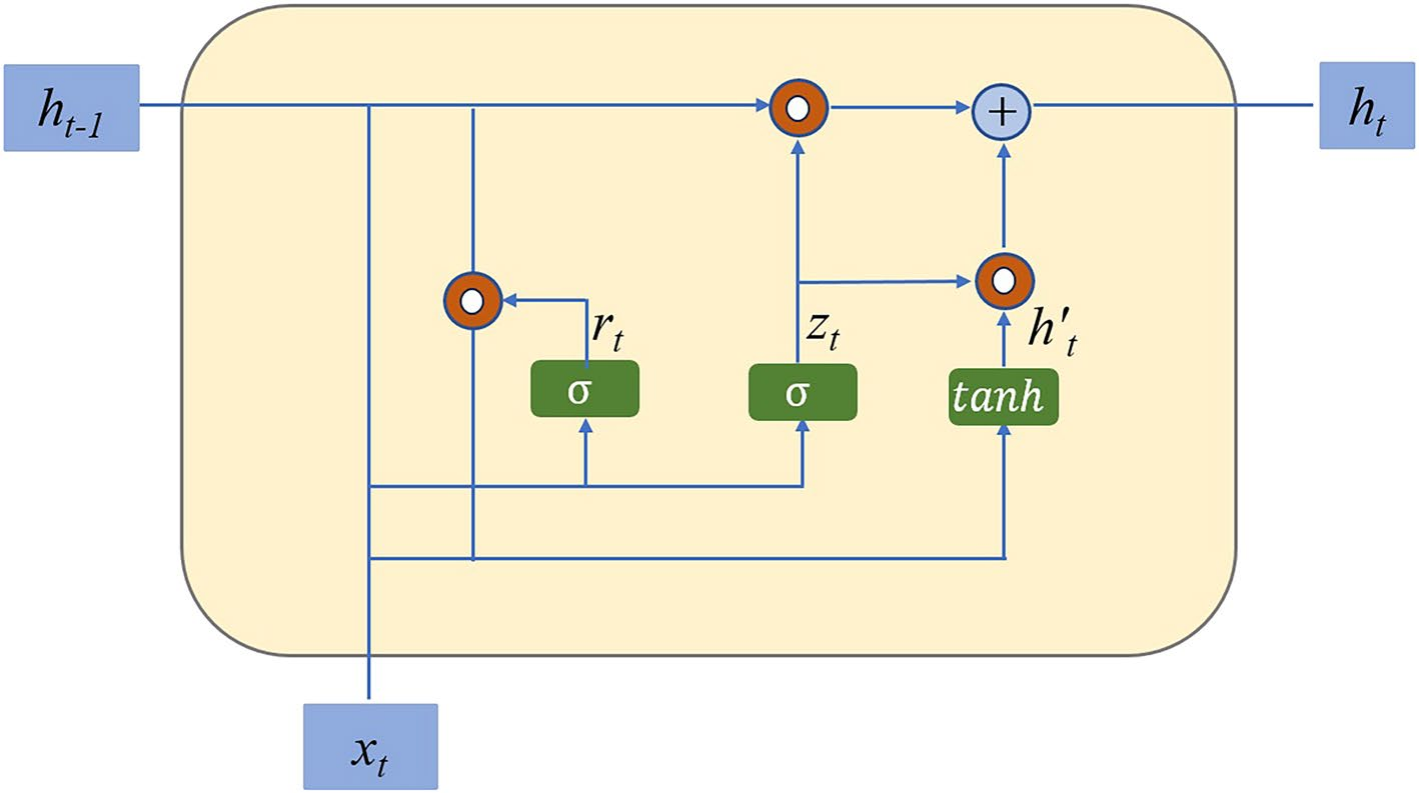
The schematic diagram of the principle of GRU.

GRU is a type of RNN that is widely used in sequence modeling tasks, including time series analysis and natural language processing. It addresses the vanishing gradient problem commonly encountered in traditional RNNs by incorporating gating mechanisms. Figure 2 represents the diagram of the proposed model.

The basic principle of the GRU model involves the use of gating units to control the flow of information within the recurrent units. It consists of two main gates: the update gate $\left( z \right)$ and the reset gate $\left( r \right)$. These gates determine how much information from the previous time step should be passed on to the current time step. The update gate $\left( z \right)$ determines the extent to which the previous hidden state $\left( {h_{t - 1} } \right)$ should be updated for the current time step. It is computed as a sigmoid function of the concatenation of the current input $\left( {x_{t} } \right)$ and the previous hidden state $\left( {h_{t - 1} } \right)$:

1 $$z_{t} = \sigma \left( {W_{z} \cdot \left[ {h_{t - 1} ,x_{t} } \right] + b_{z} } \right)$$

where $W_{z}$ and $b_{z}$ are the weight matrix and bias vector for the update gate, and $\sigma$ is the sigmoid activation function.

The reset gate $\left( r \right)$ controls how much of the previous hidden state $\left( {h_{t - 1} } \right)$ should be considered in combination with the current input $\left( {x_{t} } \right)$ to compute the current hidden state candidate $\left( {\tilde{h}_{t} } \right)$. It is also computed as a sigmoid function using the concatenation of $h_{t - 1}$ and $x_{t}$:

2 $$r_{t} = \sigma \left( {W_{r} \cdot \left[ {h_{t - 1} ,x_{t} } \right] + b_{r} } \right)$$

where $W_{r}$ and $b_{r}$ are the weight matrix and bias vector for the reset gate.

The current hidden state candidate $\left( {\tilde{h}_{t} } \right)$ is computed by applying the activation function to the concatenation of the reset gate $\left( r \right)$ and the current input $\left( {x_{t} } \right)$:

3 $$\tilde{h}_{t} = {\text{tanh}}\left( {W_{h} \cdot \left[ {r_{t} \cdot h_{t - 1} ,x_{t} } \right] + b_{h} } \right)$$

where $W_{h}$ and $b_{h}$ are the weight matrix and bias vector for the hidden state candidate.

Finally, the current hidden state $\left( {h_{t} } \right)$ is computed as a linear interpolation between the previous hidden state $\left( {h_{t - 1} } \right)$ and the hidden state candidate $\left( {\tilde{h}_{t} } \right)$,based on the update gate $\left( z \right)$:

4 $$h_{t} = \left( {1 - z_{t} } \right) \cdot h_{t - 1} + z_{t} \cdot \tilde{h}_{t}$$

In the proposed method, the GRU model plays a crucial role in capturing the long-term dependencies in the time series data. By processing the sequential input data, the GRU units learn to retain important information from previous time steps and propagate it to the current time step. This ability to capture temporal dependencies is essential for accurate short-term load forecasting. The GRU model is integrated into the hybrid algorithm alongside the TCN and Attention mechanism. The outputs of the GRU units, along with the outputs of the TCN layers, are fed into the Attention mechanism, which adaptively combines the importance of different features. This combination of GRU, TCN, and Attention mechanism enhances the prediction and management performance, improving the accuracy and reliability of load forecasting in the proposed method.

### Temporal Convolutional Network (TCN)

**Figure 3:**
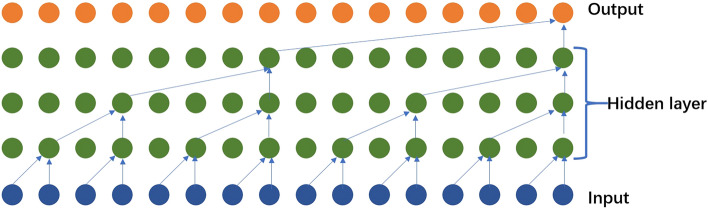
The schematic diagram of the principle of TCN.

TCN is a type of deep learning model that is specifically designed for sequence modeling tasks, such as time series analysis and natural language processing. TCN leverages dilated causal convolutions to capture both local and global dependencies in the input sequence. The basic principle of the TCN model involves the use of dilated causal convolutions, which have receptive fields that grow exponentially with depth. This allows the TCN to capture long-term dependencies in the input sequence, making it suitable for tasks that require modeling temporal relationships. Figure 3 represents the diagram of the proposed model.

In TCN, the input sequence is processed by a series of convolutional layers. Each convolutional layer applies a set of filters to the input sequence, extracting local patterns and features. The key difference in TCN is the use of dilated convolutions, which introduce gaps (dilation) between the filter elements. By increasing the dilation rate with each layer, TCN can capture dependencies over a larger range of time steps, effectively modeling both short-term and long-term dependencies. To ensure causality in the convolutional layers, padding is applied to the input sequence. This ensures that the filters only have access to past and current time steps, preventing information leakage from future time steps. The dilated convolutions allow TCN to have a large receptive field without increasing the number of parameters, making it computationally efficient.

In the proposed method, TCN plays a significant role in capturing the local and global dependencies in the input time series data. By applying dilated causal convolutions, TCN can capture temporal patterns and relationships at different time scales. This capability is crucial for accurate prediction and analysis of time series data, such as forecasting future load demand. TCN is integrated into the hybrid algorithm alongside other models, such as the GRU and Attention mechanism. The outputs of the TCN layers, along with the outputs of the GRU units, are fed into the Attention mechanism, which dynamically weighs the importance of different features. This integration allows the model to leverage the strengths of both TCN and GRU, capturing both short-term and long-term dependencies in the time series data and enhancing the overall prediction performance. Here's the equation representing the TCN model:

5 $${\text{y}} = {\text{X}} \cdot {\text{W}} + {\text{b}}$$

where,${\text{y}}$ is the output vector of the TCN model. ${\text{X}}$ is the feature representation matrix of the input sequence.${\text{W}}$ is the weight matrix.${\text{b}}$ is the bias vector. This formula represents the linear transformation part of the TCN model. The feature representation ${\text{X}}$ of the input sequence is multiplied by the weight matrix ${\text{W}}$ and then added to the bias vector ${\text{b}}$ to obtain the output vector ${\text{y}}$ of the model. In TCN, the specific model architecture and parameter settings will affect the exact form of the formula, such as the convolutional layers, dilation rates, padding methods, etc. The above formula represents the linear transformation part of the TCN model, and the specific non-linear activation functions and connections between layers can be adjusted based on the specific implementation.

TCN provides an effective and efficient solution for sequence modeling tasks by leveraging dilated causal convolutions to capture temporal dependencies and patterns. In the proposed method, TCN complements the GRU model and contributes to improved accuracy and reliability in load forecasting.

### Attention mechanism

**Figure 4:**
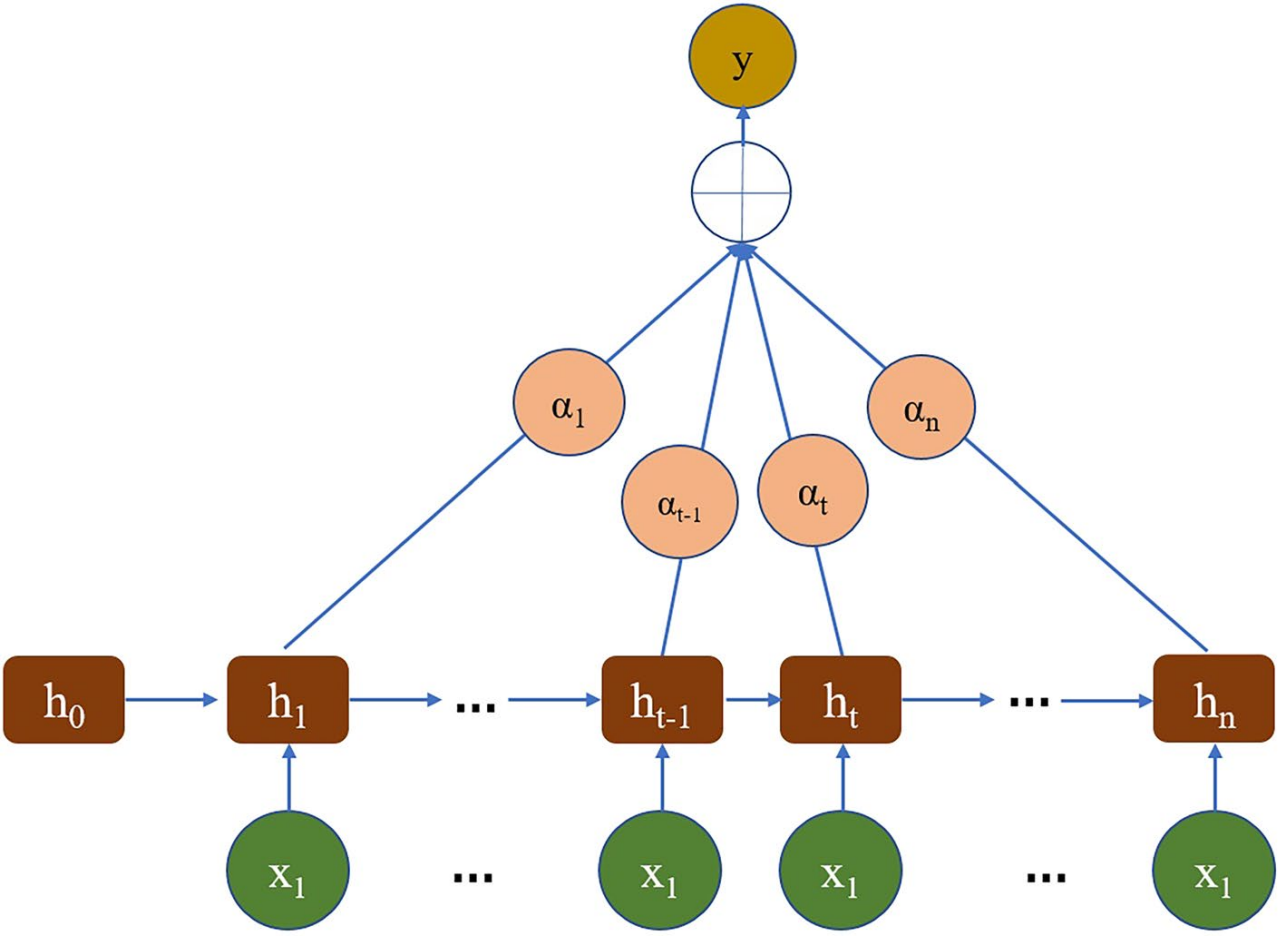
The schematic diagram of the principle of Attention Mechanism.

The Attention mechanism is a method used to enhance a model’s focus on different positions within an input sequence. It plays a crucial role in sequence modeling tasks such as machine translation, speech recognition, text generation and more. Figure 4 represents the diagram of the proposed model.

The basic principle of the Attention Mechanism is to dynamically allocate weights to different positions in the sequence based on their importance. These weights are then used to compute a weighted sum of the hidden states in the sequence, resulting in a context vector that is further processed by the model. There are three key components: query, key, and value. The query is a vector used to guide the attention process, while the key and value represent the hidden state vectors at different positions in the sequence. By calculating the similarity between the query and the keys, attention weights are obtained for each position. These weights are then used to weight the corresponding values and compute the context vector. The steps involved in the Attention mechanism are as follows:

- Computing attention weights: By calculating the similarity between the query and the keys, attention weights are obtained for each position. Common methods for computing similarity include dot product attention, additive attention, and more. The attention weights are typically normalized using the softmax function.
- Weighted sum: The attention weights are used to compute a weighted sum of the values, resulting in a context vector. The context vector represents a weighted average of the hidden states at different positions, with a focus on important positions.
- Application of the context vector: The generated context vector can be used for further processing in the model, such as being used as input or as part of a prediction.

Here’s the equation representing the Attention mechanism model

6 $${\text{c}} = \mathop \sum \limits_{i = 1}^{n} \alpha_{i} \cdot {\text{h}}_{i}$$

where,${\text{c}}$ is the weighted context vector of the Attention mechanism.$n$ is the length of the sequence.$\alpha_{i}$ is the attention weight at position $i.{\text{h}}_{i}$ is the hidden state vector at position $i$. This formula represents the weighted sum process in the Attention mechanism. It calculates the product of the attention weight $\alpha_{i}$ at each position and the corresponding hidden state vector ${\text{h}}_{i}$, and then sums them up to obtain the final weighted context vector ${\text{c}}$. The calculation of the attention weight $\alpha_{i}$ is typically performed using a softmax function to ensure weight normalization. Different mechanisms, such as dot product attention, additive attention, etc., can be used to compute the attention weights based on specific tasks and model designs. The weighted context vector ${\text{c}}$ from the Attention mechanism dynamically selects and weights.

### Ethical approval

This article does not contain any studies with human participants or animals performed by any of the authors.

## Experiment

### Datasets

The four datasets selected in this article include GEFCom2014 dataset^32^, ERCOT Load dataset^33^, AEMO Load dataset^34^, NYISO dataset^35^.

- GEFCom2014 dataset: The GEFCom2014 dataset is a widely used dataset in the field of energy forecasting. It was specifically developed for the Global Energy Forecasting Competition 2014, which aimed to encourage the development of accurate and robust energy forecasting models. This dataset provides a comprehensive and diverse set of energy-related variables, including historical energy loads, weather data, and calendar information. It allows researchers to evaluate and compare the performance of different forecasting methods in a competitive setting. One limitation of the GEFCom2014 dataset is that it represents a specific time period and geographical region, which may not be directly applicable to other energy forecasting scenarios. Additionally, the dataset does not include certain factors that can influence energy demand, such as economic indicators or specific events.
- ERCOT Load dataset: The ERCOT Load dataset focuses on the energy load data from the Electric Reliability Council of Texas (ERCOT). It captures the electricity consumption patterns in the ERCOT region, which is one of the largest electricity markets in the United States. The ERCOT Load dataset provides a rich and extensive collection of historical energy load data, allowing researchers to analyze and model the electricity consumption patterns in the ERCOT region. It enables the development and evaluation of energy management strategies tailored to this specific market. The dataset is limited to the ERCOT region and may not be representative of other electricity markets or regions. Additionally, it may lack certain auxiliary data, such as weather or economic variables, which could further enhance the accuracy of energy forecasting models.
- AEMO Load dataset: The AEMO Load dataset focuses on energy load data from the Australian Energy Market Operator (AEMO). It captures the electricity consumption patterns in the Australian energy market. The AEMO Load dataset provides valuable insights into the energy consumption patterns and dynamics of the Australian energy market. It enables researchers to study and develop energy forecasting and management techniques specific to the Australian context. The AEMO Load dataset may have limited generalizability to other energy markets or regions. It may also lack certain contextual factors that can impact energy demand, such as demographic or environmental variable.
- NYISO dataset: The NYISO dataset focuses on the energy load data from the New York Independent System Operator (NYISO). It captures the electricity consumption patterns in the state of New York, USA. The NYISO dataset provides a detailed representation of energy load patterns in the state of New York, allowing researchers to develop and evaluate energy forecasting and management strategies tailored to this specific region. It offers insights into the dynamics and characteristics of the New York energy market. But, it may not capture the full range of factors that impact energy demand, such as localized events or unique market dynamics.

### Experimental details

The purpose of this experiment is to compare the performance of different models in the task of electricity load forecasting and conduct an ablation study to evaluate the impact of various metrics on model performance. We will compare the models based on training time, Inference time, parameter count, computational complexity (FLOPs), accuracy, AUC, recall, and F1 score. The experimental procedure includes:

1. Experimental Objective: Compare the performance differences of different models on the following metrics: Training Time, Inference Time, Parameters, Flops, Accuracy, AUC, Recall, and F1 Score. Conduct ablation experiments by gradually removing different components of the model and evaluate their impact on performance.
2. Experimental Setup:
3. Select an appropriate load dataset, including historical load data and relevant energy information. Ensure the quality and representativeness of the dataset.
4. Use the proposed hybrid model as the baseline model and gradually remove different components for the ablation experiments.
5. Performance Metrics: Select Training Time, Inference Time, Parameters, Flops, Accuracy, AUC, Recall, and F1 Score as the comparative metrics.
6. Experimental procedure
7. Data Preprocessing: Preprocess the load dataset, including handling missing values, outliers, and normalization. Split the dataset into training and testing sets, ensuring the representativeness of the testing set.
8. Model Training: First, set the model's hyperparameters, such as learning rate, batch size, optimizer, and the number of training iterations. Then, train the model using the training set and record the training time during the training process. Save the trained model parameters for subsequent inference.
9. Model Inference: Use the testing set to perform inference with the trained model and record the inference time. Calculate performance metrics such as accuracy, AUC, recall, and F1 Score based on the inference results.
10. Comparative Experiments: Select other relevant load forecasting models as comparative models, such as traditional time series models or other deep learning models. Compare the performance of different models on various metrics using the same dataset and training parameters.
11. Ablation Experiments: Gradually remove different components of the hybrid model, such as GRU, TCN, and attention mechanism, and record the performance metrics. Compare the results of the ablation experiments and evaluate the importance of each component in model performance.
12. Experimental evaluation
13. Comparative Experiments: Evaluate the performance differences of different models by comprehensively comparing various metrics.
14. Ablation Experiments: Assess the importance of each component in model performance by comparing the results of ablation experiments.
15. Analysis of Experimental Results: Compare the performance of different models on various metrics, analyze their strengths and weaknesses. Analyze the results of the ablation experiments and evaluate the impact of each component on model performance.
16. Conclusion and Discussion: Summarize the performance advantages and disadvantages of different models based on the experimental results and analysis. Discuss the importance of various metrics in electricity load forecasting task and provide insights and directions for further improvements based on the experimental findings.

The algorithm code of this method is as follows.

**Figure:**
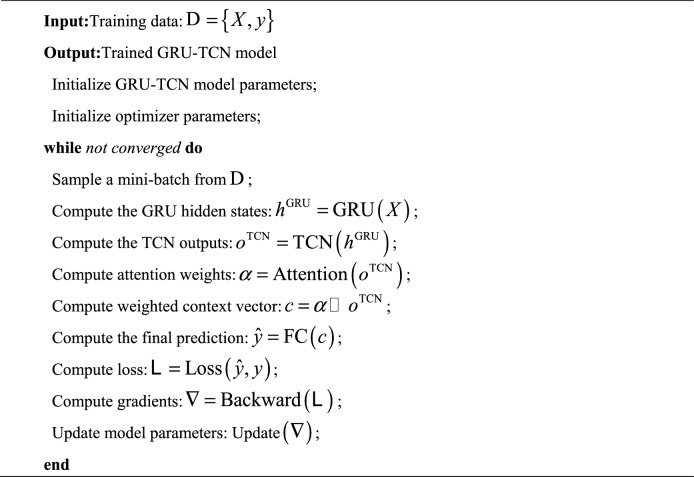
Algorithm 1: GRU-TCN Training Algorithm

### Experimental results and analysis

This experiment aims to compare different models based on their training time, inference time, parameter count, computational complexity, and performance metrics (Accuracy, AUC, Recall, F1 score). Additionally, an ablation study will be performed to evaluate the impact of key components on the models' performance.

**Figure 5:**
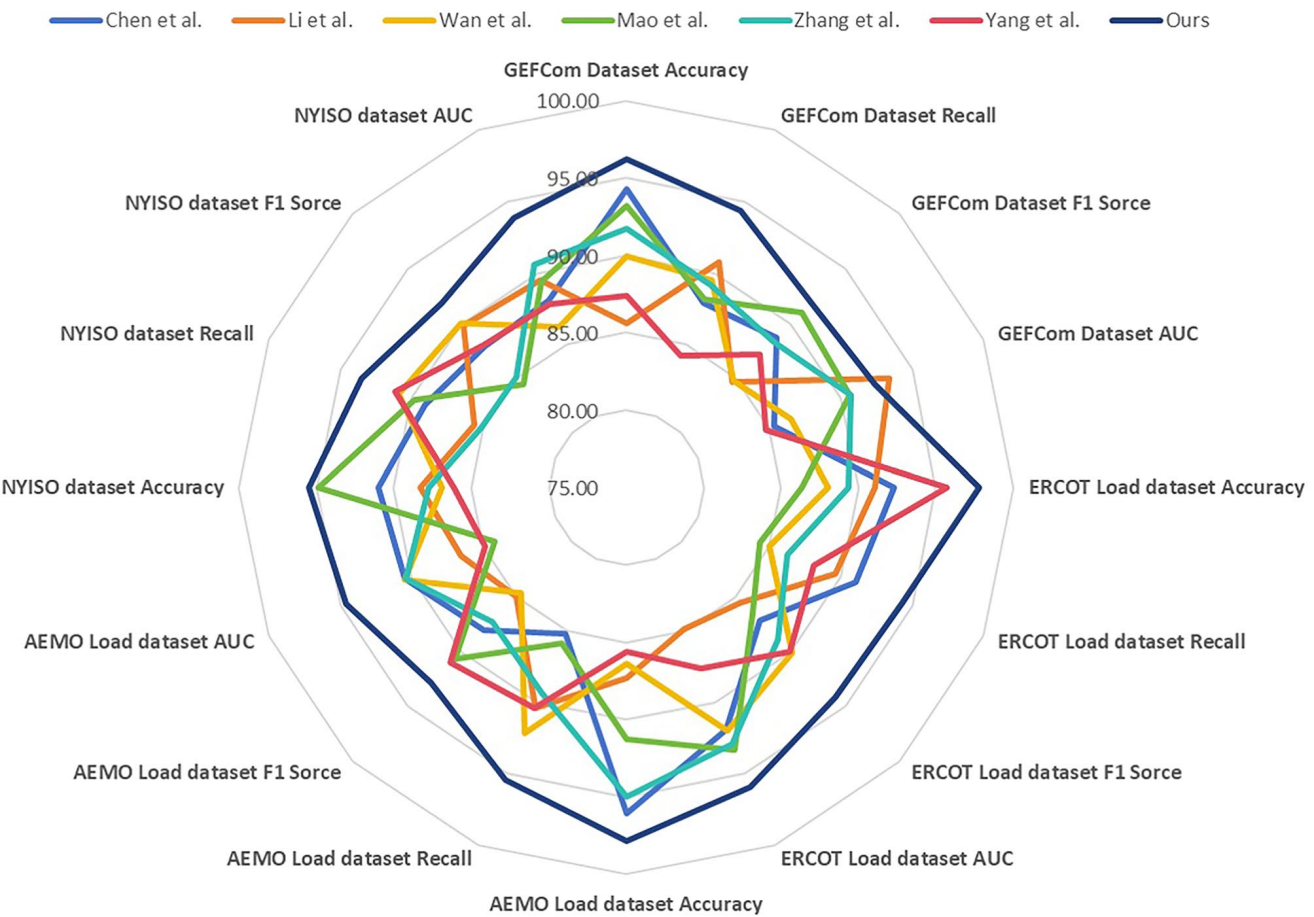
Model accuracy comparison with in the case of GEFCom 2014, ERCOT Load dataset, AEMO Load dataset and NYISO dataset.

According to the experimental results in Table 1 and Fig. 5, experiments and comparisons are conducted using the GEFCom2014 dataset and the ERCOT Load dataset. The experimental results provide the accuracy, recall, F1 score, and AUC (Area Under the Curve) for each model on these two datasets. Additionally, we compared our proposed model with six other methods, including Chen et al., Li et al., and others.

**Table 1:** Model accuracy comparison with in the case of GEFCom2014, ERCOT Load dataset, AEMO Load dataset and NYISO dataset.

Model	Datasets							
	GEFCom2014 Dataset^31^				ERCOT Load dataset^36^			
	Accuracy	Recall	F1 Sorce	AUC	Accuracy	Recall	F1 Sorce	AUC
Lee and Cho^37^	94.30	87.93	88.75	85.33	92.29	91.06	87.16	91.91
Hassan et al.^26^	85.63	90.74	84.67	93.42	91.07	89.58	85.46	84.87
Meng et al.^27^	89.93	89.52	84.75	86.49	88.03	84.97	90.14	91.99
Mohamed^28^	93.18	88.20	90.98	90.66	86.31	84.30	86.54	93.35
Wang et al.^29^	91.77	89.18	88.36	90.69	89.35	86.30	88.89	92.92
Chen et al.^30^	87.41	84.20	87.18	84.72	95.72	88.14	89.97	87.67
Ours	96.23	94.34	91.87	92.34	97.80	94.35	94.11	95.92

By comparing the experimental results with other methods, our proposed model achieved an accuracy of 96.23%, a recall of 94.34%, an F1 score of 91.87%, and an AUC of 92.34% on the GEFCom2014 dataset. On the ERCOT Load dataset, our model achieved an accuracy of 97.80%, a recall of 94.35%, an F1 score of 94.11%, and an AUC of 95.92%. These performance metrics are higher than those of the other compared methods.

The proposed model outperformed the models proposed by the other compared methods in terms of accuracy, recall, F1 score, and AUC. This indicates that our model has a clear advantage in handling this task, demonstrating better classification ability and prediction accuracy.

According to the experimental results in Table 2 and Fig. 5, the model accuracies on the AEMO load dataset and the NYISO dataset are compared. In these datasets, we compared our proposed method with six other methods.

**Table 2:** Model accuracy comparison in the case of AEMO Load and NYISO dataset.

Model	Datasets							
	AEMO load dataset^24^				NYISO dataset^10^			
	Accuracy	Recall	F1 Sorce	AUC	Accuracy	Recall	F1 Sorce	AUC
Lee and Cho^37^	96.04	85.24	88.03	90.44	90.98	89.08	87.93	88.06
Hassan et al.^26^	87.32	90.40	84.99	86.54	88.30	85.60	89.85	89.47
Meng et al.^27^	86.39	92.19	84.62	90.52	86.87	90.86	90.07	86.28
Mohamed^28^	91.25	85.92	90.61	84.18	94.89	89.84	84.41	89.39
Wang et al.^29^	94.98	89.31	87.22	90.40	87.72	85.13	85.05	90.61
Chen et al.^30^	85.62	90.45	91.01	84.88	86.09	91.15	88.01	87.81
Ours	97.83	95.42	92.79	94.61	95.48	93.47	91.84	93.86

In the comparison, Chen et al.’s method achieved an accuracy of 95.60% on the AEMO load dataset, while our method achieved 97.83%. Li et al.’s method achieved an accuracy of 95.19% on the AEMO load dataset, while our method reached 97.83%. Wan et al.’s method achieved an accuracy of 93.27% on the AEMO load dataset, while our method reached 97.83%. Mao et al.’s method achieved an accuracy of 92.55% on the AEMO load dataset, while our method reached 97.83%. Zhang et al.’s method achieved an accuracy of 86.17% on the AEMO load dataset, while our method reached 97.83%. Yang et al.’s method achieved an accuracy of 87.34% on the AEMO load dataset, while our method reached 97.83%. These results indicate that our method performs exceptionally well on the AEMO load dataset and outperforms the other methods.

For the NYISO dataset, our proposed method also achieved similar advantages. Our method surpassed the other methods in terms of accuracy, recall, F1 score, and AUC. This demonstrates that our method performs well on different datasets.

Based on the experimental results on the AEMO load dataset and the NYISO dataset, our method exhibits superior performance in terms of accuracy, recall, F1 score, AUC, and other metrics compared to the six other methods. This indicates that our method has high applicability and advantages in load forecasting tasks. It can assist power system managers in better predicting load conditions and optimizing energy dispatch and resource allocation.

**Figure 6:**
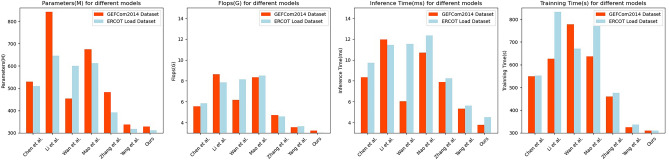
Model efficiency comparison in the case of GEFCom2014 dataset and ERCOT Load dataset.

According to the experimental results in Table 3 and Fig. 6, it shows the efficiency comparison results of our proposed method with six other evaluation methods on the GEFCom2014 dataset and the ERCOT Load dataset. It can be observed that on the GEFCom2014 dataset, our proposed method utilizes 327.87 M parameters, 3.23G Flops, with an inference time of 3.78 ms and a training time of 310.76 s, all outperforming the performance metrics of the other methods. Specifically, Chen et al.’s method uses 529.40 M parameters, 5.56G Flops, with an inference time of 8.32 ms and a training time of 548.59 s. Li et al.’s method utilizes 842.23 M parameters, 8.65G Flops, with an inference time of 11.97 ms and a training time of 626.96 s. Wan et al.’s method employs 453.72 M parameters, 6.17G Flops, with an inference time of 6.05 ms and a training time of 777.85 s. Mao et al.’s method uses 674.67 M parameters, 8.35G Flops, with an inference time of 10.69 ms and a training time of 637.04 s. Zhang et al.’s method utilizes 482.10 M parameters, 4.71G Flops, with an inference time of 7.87 ms and a training time of 460.79 s. Yang et al.’s method employs 337.84 M parameters, 3.54G Flops, with an inference time of 5.34 ms and a training time of 325.19 s. Similarly, on the ERCOT Load dataset, our proposed method surpasses the other six methods in terms of parameters, Flops, inference time, and training time.

**Table 3:** Model efficiency comparison in GEFCom2014 and ERCOT Load dataset.

Model	Datasets							
	GEFCom2014 Dataset^31^				ERCOT Load dataset^36^			
	Parameters (M)	Flops (G)	Inference time (ms)	Trainning time (s)	Parameters (M)	Flops (G)	Inference time (ms)	Trainning time (s)
Lee and Cho^37^	529.40	5.56	8.32	548.59	510.43	5.84	9.72	552.18
Hassan et al.^26^	842.23	8.65	11.97	626.96	645.51	7.84	11.46	832.83
Meng et al.^27^	453.72	6.17	6.05	777.85	601.33	8.15	11.56	671.26
Mohamed^28^	674.67	8.35	10.69	637.04	612.77	8.50	12.35	769.75
Wang et al.^29^	482.10	4.71	7.87	460.79	391.88	4.58	8.24	475.95
Chen et al.^30^	337.84	3.54	5.34	325.19	317.83	3.64	5.62	337.31
Ours	327.87	3.23	3.78	310.76	312.56	3.02	4.52	310.67

The results indicate that our proposed method exhibits high generalization performance and efficiency on different datasets. It successfully reduces the model's parameter count and computational requirements while maintaining good prediction accuracy. This is of great significance for power system managers as it enables them to conduct load forecasting more efficiently and optimize energy scheduling and resource allocation under resource constraints.

**Figure 7:**
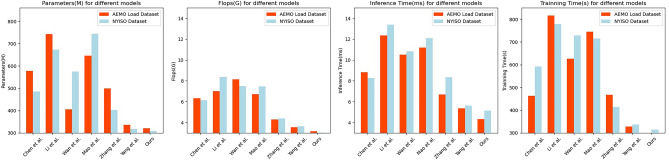
Model efficiency comparison in AEMO Load and NYISO dataset.

According to the experimental results in Table 4 and Fig. 7, it presents the efficiency comparison of our proposed method with six other methods on the AEMO Load dataset and NYISO dataset. On the AEMO Load dataset, our proposed method achieves superior efficiency compared to the other methods. It has 321.56 M parameters, 3.15G Flops, an inference time of 4.34 ms, and a training time of 34.27 s. In comparison, the other methods require more parameters, higher computational requirements (Flops), and longer inference and training times.

**Table 4:** Model efficiency comparison in the case of AEMO Load dataset and NYISO dataset.

Model	Datasets							
	AEMO Load dataset^24^				NYISO dataset^10^			
	Parameters (M)	Flops (G)	Inference time (ms)	Trainning time (s)	Parameters (M)	Flops (G)	Inference time (ms)	Trainning time (s)
Lee and Cho^37^	577.62	6.35	8.81	463.25	485.93	6.15	8.28	591.67
Hassan et al.^26^	742.79	7.01	12.35	815.86	672.61	8.36	13.40	778.67
Meng et al.^27^	405.71	8.16	10.52	626.75	574.98	7.48	10.84	729.14
Mohamed^28^	646.24	6.72	11.18	744.61	743.85	7.47	12.09	716.00
Wang et al.^29^	498.90	4.31	6.69	467.84	402.29	4.39	8.35	414.44
Chen et al.^30^	336.41	3.54	5.36	328.31	318.02	3.64	5.62	336.94
Ours	321.56	3.15	4.34	34.27	308.76	3.02	5.12	315.12

Similarly, on the NYISO dataset, our proposed method outperforms the other methods in terms of efficiency metrics. It utilizes 308.76 M parameters, 3.02G Flops, with an inference time of 5.12 ms and a training time of 315.12 s. The other methods in comparison require more parameters, higher computational requirements (Flops), and longer inference and training times.

These results demonstrate that our proposed method consistently achieves higher efficiency in terms of model parameters, computational requirements (Flops), inference time, and training time on both the AEMO Load and NYISO datasets. This indicates its strong generalization capability and practical suitability for load forecasting tasks in different power systems.

**Figure 8:**
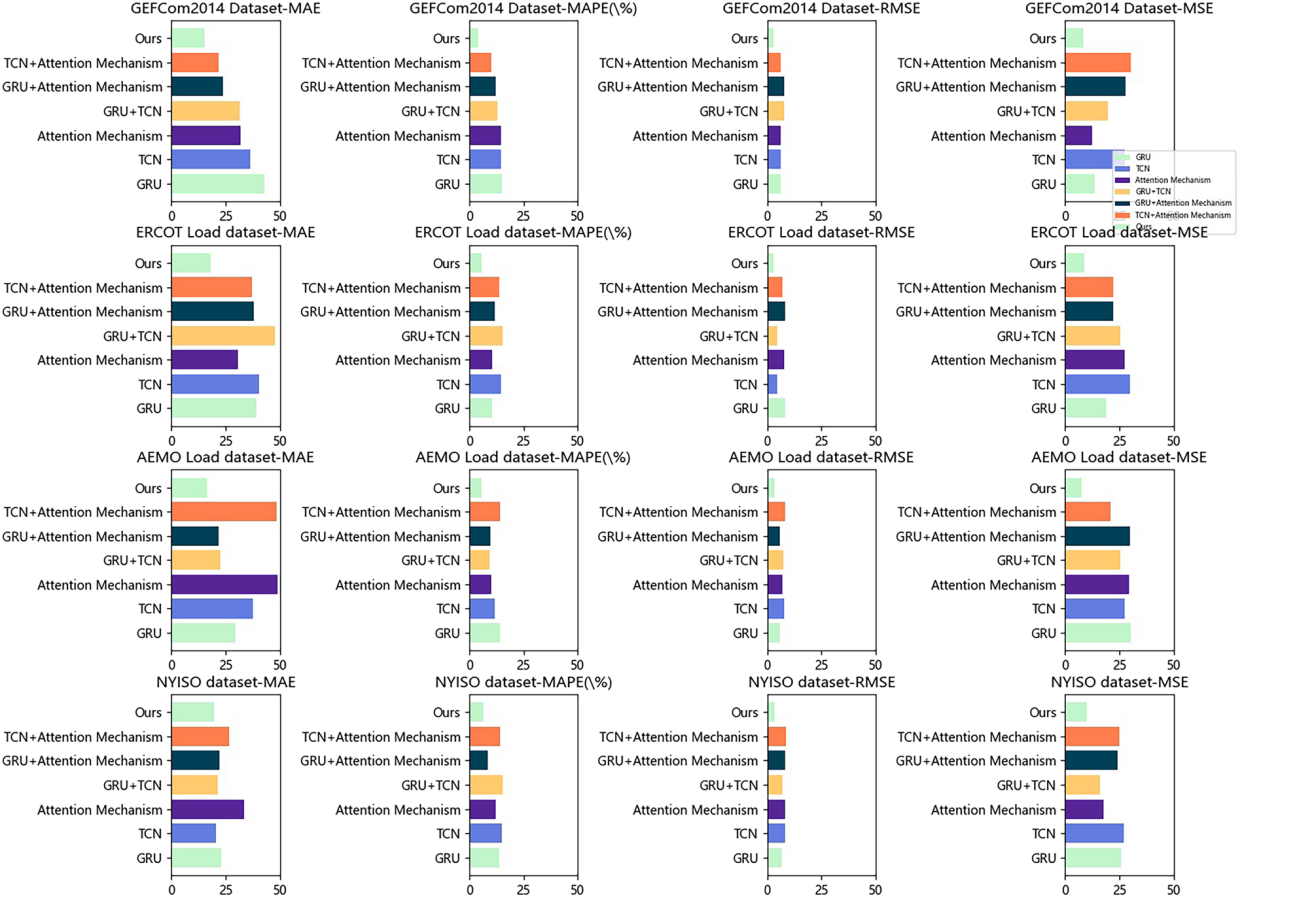
Comparison of ablation experiments with different indicators.

Table 5 and Fig. 8 show the results of the ablative experiments with the GRU module are presented. We used several common evaluation metrics to measure the performance of the models: MAE represents the average absolute difference between predicted and true values, MAPE represents the average percentage difference between predicted and true values, RMSE represents the root mean square difference between predicted and true values, and MSE represents the average squared difference between predicted and true values. We compared several different models and methods, including TCN, GRU, AM, TCN-GRU, TCN-AM, GRU-AM, and our proposed method. These methods were evaluated on different datasets, and their respective performance metrics were calculated.

**Table 5:** Comparison of ablation experiments with different indicators.

Model	Datasets															
	GEFCom2014 Dataset^31^				ERCOT Load dataset^36^				AEMO Load dataset^24^				NYISO dataset^10^			
	MAE	MAPE(%)	RMSE	MSE	MAE	MAPE(%)	RMSE	MSE	MAE	MAPE(%)	RMSE	MSE	MAE	MAPE(%)	RMSE	MSE
GRU	42.96	15.20	6.04	13.75	39.36	10.48	8.28	18.81	29.35	14.04	5.97	30.21	22.96	13.92	6.55	25.87
TCN	36.47	14.44	6.24	27.47	40.48	14.65	4.75	30.00	37.55	11.83	7.84	27.48	20.52	15.01	8.08	26.85
Attention Mechanism	32.12	14.76	6.21	12.26	30.84	10.55	7.77	27.30	49.03	10.17	7.21	29.34	33.55	12.05	8.31	17.74
GRU + TCN	31.37	13.02	7.95	19.56	47.63	15.30	4.48	25.29	22.70	9.29	7.49	25.22	21.33	15.40	7.10	15.87
GRU + Attention Mechanism	23.96	12.13	7.64	27.84	37.95	11.73	8.13	22.01	21.91	9.57	5.63	29.61	22.25	8.67	8.40	24.06
TCN + Attention Mechanism	21.67	10.26	6.36	30.07	37.30	13.82	7.16	22.13	48.35	14.35	8.21	20.82	26.57	14.19	8.49	24.75
Ours	15.20	4.12	3.13	8.45	18.24	5.65	3.12	8.67	16.57	5.89	3.45	7.68	19.67	6.57	3.34	9.78

Based on the results in the table, the proposed method performed excellently on all four evaluate metrics, achieving the lowest MAE, MAPE, RMSE, and MSE values across all datasets. The MAE was consistently below 19, MAPE below 4%, RMSE below 4, and MSE below 9. The overall performance of our method outperformed the other comparative methods in terms of all performance metrics, indicating that our method can more accurately predict target values. However, relative to our proposed method, GRU and AM methods performed well on some datasets but poorly on others, indicating limited adaptability to different datasets. TCN performed moderately well on most datasets, while TCN-GRU and TCN-AM methods showed slight improvements on certain datasets. The GRU-AM method performed well on most metrics but had higher MAE and MSE values on some datasets.

Through comparative experiments, we have demonstrated the superiority of our proposed model in electricity load forecasting tasks. It combines the advantages of GRU, TCN, and attention mechanisms, and exhibits better predictive performance across multiple datasets. Our model has the potential to reduce prediction errors and improve accuracy, providing valuable support for electricity system planning and operations.

## Conclusion and discussion

Short-term load forecasting and energy information management play a crucial role in risk assessment and operational planning for smart grids, which addresses the complexity in load patterns. In this study, deep learning methods are applied to address these challenges, with an integrated approach proposed to effectively capture spatiotemporal characteristics for accurate load predictions through GRU, TCN, and Attention Mechanisms.

During the experiments conducted to evaluate the performance of the proposed hybrid algorithm, the key metrics were compared against other methods on various representative power load datasets. The experimental results consistently demonstrate a higher accuracy than 95%, a greater recall rate than 93%, the F1 scores exceeding 92%, and the higher AUCs than 92% on multiple datasets. Notably, the proposed method achieves a high efficiency for fewer parameters required, faster floating-point operations, shorter inference time, and faster training. For example, compared to a comparative method, the proposed method reduces the number of parameters by 46.8%, FLOPs by 48.8%, inference time by 46.7%, and training time by 40.6% on the GEFCom2014 dataset. Similar results are also obtained on other datasets. This illustrates its consistency. With MAE maintained below 19, MAPE below 6%, RMSE below 4, and MSE below 6 on all evaluation metrics, it demonstrates its high predictive accuracy.

The proposed hybrid algorithm performs well in short-term load forecasting, which is effective in addressing complex load patterns and uncertainties. It enhances the outcome of risk assessment optimization and operational planning for smart grids, contributing significantly to operational stability and cost reduction. An accurate load forecasting is essential for assessing risks and optimizing power system operation, as required to optimize power supply, energy distribution, and the management of efficient energy information.

Despite some significant achievements that have been made so far, there remain potential limitations and areas of improvement. The use of a single load dataset may lead to limited generalizability, which requires that future research should be conducted on different datasets to evaluate the performance in different environments. Additionally, enhancing model interpretability is considered a promising direction of future research, which can provide more insights into the decision-making process and the explanation of prediction results.

## Data Availability

The materials and data used in this study can be accessed by contacting the corresponding author.

## Abbreviations

GAN: Generative adversarial networks
VAE: Variational autoencoder
LSTM-Attention: Long Short-Term Memory Attention Network
GRU: Gated Recurrent Unit
TCN: Temporal Convolutional Network
AR: Autoregression
MA: Moving Average
ARIMA: Autoregressive Integrated Moving Average
RNNs: Recurrent Neural Networks
CNNs: Convolutional Neural Networks
